# Bacterial meningitis in children with an abnormal craniocerebral structure

**DOI:** 10.3389/fped.2023.997163

**Published:** 2023-03-28

**Authors:** Jiali Pan, Wei Xu, Wenliang Song, Tao Zhang

**Affiliations:** Department of Pediatrics, ShengJing Hospital of China Medical University, Shenyang, China

**Keywords:** bacterial meningitis, abnormal craniocerebral structure, pediatric intensive care unit, children, infection

## Abstract

**Background:**

We studied the causative pathogens, clinical characteristics, and outcome of bacterial meningitis in children with an abnormal craniocerebral structure.

**Methods:**

A retrospective single-center study was conducted on children aged in the range of 29 days to 14 years by using data obtained from the pediatric intensive care unit in Shengjing Hospital between January 2014 and August 2021. All children were diagnosed with bacterial meningitis. They were divided into complex and simple groups by taking into account the presence of an abnormal craniocerebral structure before they contracted bacterial meningitis. We collected data on demographics, clinical presentations, laboratory results, imaging studies, treatments, and outcomes.

**Results:**

A total of 207 patients were included in the study (46 in the complex group and 161 in the simple group). Patients in the complex group had a lower mortality rate (6.5% vs. 11.2%, *p* < 0.05), positive blood culture (13.0% vs. 34.8%; *p* < 0.05), multiple organ dysfunction syndrome (0% vs. 9.3%; *p* < 0.05), and shock (2.2% vs. 9.3%; *p* = 0.11). These patients were more often detected with neurological sequelae (80.4% vs. 53.4%; *p* < 0.05), cerebrospinal fluid drainage (50% vs. 15.5%; *p* < 0.05), nosocomial infection (54.3% vs. 3.1%; *p* < 0.05), and multidrug-resistant bacteria (62.5% vs. 55.6%, *p* = 0.501). In patients in the simple group, infection was mostly confined to the nervous system.

**Conclusion:**

Bacterial meningitis patients with an abnormal craniocerebral structure had fewer bloodstream infections, lower mortality rates, and higher incidence rates of neurological sequelae. Pathogens were more likely to be nosocomial and multidrug-resistant bacteria.

## 1. Introduction

Acute bacterial meningitis (BM) is defined as an inflammation of the meninges in response to bacterial infection (1). In the past four decades, the epidemiology of BM has changed with the presence of antiretroviral treatments of human immunodeficiency virus infection and the introduction of conjugate vaccines (2). However, the number of BM cases has increased worldwide (3). The most common causes of acute BM in China are *Streptococcus pneumoniae* and *Neisseria meningitidis* (4). In developing countries, the incidence of BM varies between 20 and 80 cases per 100,000 population, representing a substantial disease burden (5). Approximately 3% of the total number of children who are younger than 5 years die of meningitis, which is the 10th cause of death in children of this age group (5).

The risk factors for BM are unvaccinated status; immunological compromise; parameningeal infection; presence of anatomical defects in the brain, spinal cord, and inner ear; acquired skull defect; and implantation of medical devices (6). In China, universal vaccination has not been implemented, and BM is mainly caused by *S. pneumoniae*, *Haemophilus influenzae*, and *N. meningitidis* (2, 4). Most of these pathogens are community-acquired, and the craniocerebral structure is normal, with few congenital or acquired immune deficiencies (2, 4). Other pathogens causing BM have also been reported, such as *Acinetobacter baumannii* (7, 8), resistant *Enterobacteriaceae* (9), coagulase-negative *Staphylococci* (10), and *Staphylococcus aureus* (11). These pathogens are mostly observed in hospital-acquired infections, including infections as a result of neurosurgery, head trauma, skull base fractures, cerebral hemorrhage, and immunodeficiency (10). Different infection pathways have different pathogenic spectra. *S. pneumoniae*, *H. influenzae*, and *N. meningitidis* are the most common pathogens causing bloodstream infection. The incidence of bloodstream infection is related to non-vaccination during infancy and immunodeficiency (12, 13). Infection with an abnormal craniocerebral structure (ACS) mostly occurs after a disruption of the external barrier, with pathogens entering the central nervous system (CNS) by direct invasion (14). Bloodstream infection may sometimes occur. Although a contiguous source of infection (such as in cases of sinusitis or mastoiditis) is a route of bacterial entry into the CNS with a normal craniocerebral structure (NCS), the route by which pathogens cause bloodstream infection and then traverse the blood–brain barrier is more common (15). ACS can be congenital or secondarily acquired, and some examples are damage to the integrity of the skull base, the sealing structure of the skull and vertebral bodies, and the meninges, and primary or secondary hydrocephalus. Among these, the destruction of the integrity of the skull base and the sealing structure of the skull is most likely to be complicated by BM. Coagulase-negative *Staphylococci* and gram-negative Bacilli are the main pathogens causing BM associated with ACS (14), and the incidence of multidrug-resistant (MDR) organism is on the rise (16). Most previous studies have focused solely on neurosurgery, posttraumatic brain trauma, or hospital-acquired BM. This study aims to study the clinical characteristics, pathogens, treatment, and outcome of BM with ACS.

## 2. Methods

### 2.1. Study methods

**Figure 1:**
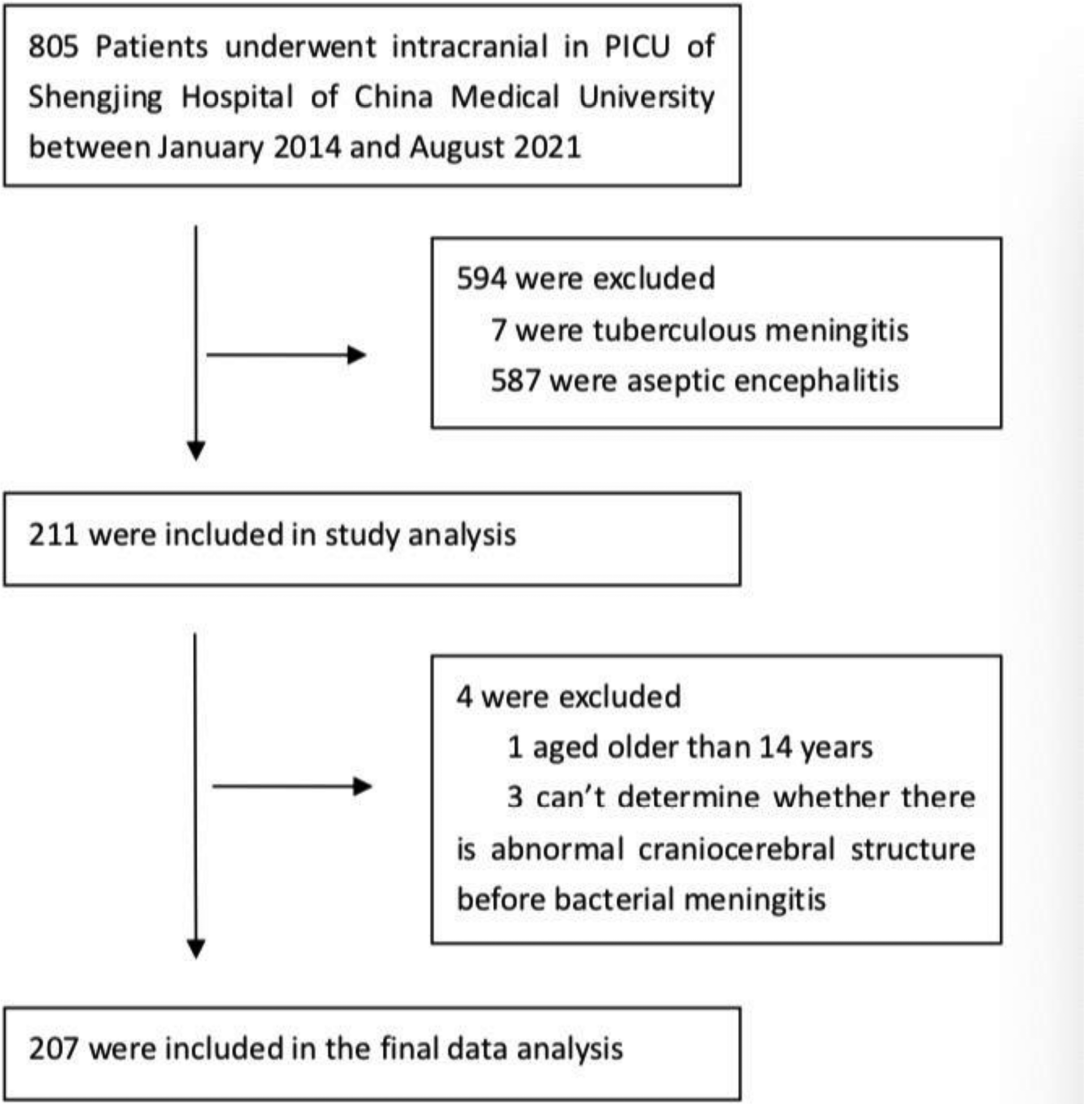
The flowchart for patient enrollment.

This was a retrospective single-center cohort study of 207 patients who were admitted in the pediatric intensive care unit (PICU) of Shengjing Hospital of China Medical University between January 2014 and August 2021. All patients were diagnosed with bacterial meningitis and were aged between 29 days and 14 years. They were divided into complex and simple groups according to the presence of ACS before the contraction of BM. Complex BM was defined as BM with ACS pre-existing the onset of infection, which is related to prior clinical diagnosis. ACS includes congenital defects in the integrity of the brain and spine, damage to the integrity of the skull base due to various reasons, damage to the sealing structures of the skull and vertebral bodies, damage to the meninges, and primary or secondary hydrocephalus. Simple BM was defined as BM without ACS. Patients were eligible to participate in the study if they met the following inclusion criteria: (1) patients diagnosed with BM, (2) patients aged 29 days to 14 years, and (3) patients whose abovementioned ACS could possibly be determined before the contraction of BM. The exclusion criteria were as follows: (1) patients diagnosed with tuberculous meningitis or aseptic encephalitis, and (2) where it was not possible to determine the presence or otherwise of ACS before BM. The flowchart for patient enrollment is given in Figure 1. Information related to demographics, symptoms and signs on admission [convulsion, Glasgow Coma Scale (GCS), shock, multiple organ dysfunction syndrome (MODS)], laboratory findings [cerebrospinal fluid (CSF), blood routine, inflammatory indicators, and pathogens] at admission and during hospitalization, clinical course, treatment (antibiotics, corticosteroids, intrathecal injection, and drainage) and neurological findings [computed tomography (CT) and magnetic resonance imaging (MRI)] at discharge, and final outcomes was collected from the medical records of the patients. During the period of treatment, the GCS score was recorded, because this is the time when it is the lowest. The CSF samples were collected for the first time with symptoms of BM. The pediatric clinical illness score (PCIS), pediatric risk of mortality III (PRISM III), blood routine, and inflammatory indicators were recorded within 24 h after the samples were obtained. Blood and CSF cultures were positive specimens at any time during hospitalization, and therefore, specimen contamination was excluded from the analysis. A head CT was done on patients within 24 h of their admission to the PICU. Patients who completed head CT or MRI examination again before discharge were examined for new or exacerbated abnormalities after neuroimaging of meningitis.

### 2.2. Diagnosis and criteria

Diagnostic criteria for BM: CSF culture is the gold standard. Children have meningitis signs and/or symptoms (fever, poor feeding, vomiting, and seizures) along with any of the following CSF changes: (1) CSF shows a glucose concentration <1.9 mmol/L, CSF glucose to blood glucose ratio of <0.23, protein concentration >2,200 mg/L, and white cell count >2,000 cells per μl (2, 17); (2) when children receive antibiotic treatment before CSF examination, CSF shows a protein concentration >400 mg/L and white blood cell counts >10 cells per μL (1); and (3) CSF PCR is positive (17).

The diagnosis of the causative pathogen is based on a positive bacterial culture or PCR of the CSF. A positive blood bacterial culture is useful for the detection of causative pathogens (17).

MDR was defined as acquired non-susceptibility to at least one agent in three or more antimicrobial categories, and extensively drug-resistant (XDR) was defined as non-susceptibility to at least one agent in all but two or fewer antimicrobial categories (18).

The site of infection was defined as an infection of the system other than the nervous system, including positive blood culture, pulmonary infection, urinary tract infection, abdominal infection, osteomyelitis, and skin infection.

The antibiotics administered during the treatment were Cephalosporins, Penicillin, Carbapenems, Glycopeptides, Oxazolidinones, Aminoglycosamines, Macrolides, Quinolones, Polymyxins, Tetracyclines, and Chloramphenicol.

Nosocomial meningitis was defined as meningitis acquired 48 h after admission (19).

Cerebral functional failure was defined as a GCS score of less than 9.

Neurological complications were defined as any new neurological symptoms or signs and abnormalities, upon a neuroimaging study, that occurred soon after an episode of meningitis or judged by a clinical neurologist to have directly resulted from an episode of meningitis.

### 2.3. Statistical analyses

The results are presented as mean ± standard deviation and median (interquartile range) and *n* (%) for continuous and categorical variables, respectively. Normality was assessed for each continuous variable. Comparisons between groups were performed using Student's *t*-test for normally distributed data and the Mann–Whitney *U*-test for non-normally distributed data. Categorical variables were tested using the chi-squared test. Statistical significance was set at *p* < 0.05. Statistical analysis was performed using the Statistical Package of the Social Sciences version 26 package program.

## 3. Results

### 3.1. Clinical characteristics

A total of 207 patients with episodes of bacterial meningitis were identified, of which 111 (53.6%) were boys. All patients were divided into two groups, complex group and simple group, on the basis of the presence of ACS or otherwise. The complex group contained 46 patients and the simple group contained 161 patients. The median age of patients was 4.0 ± 3.8 years in the complex group and 1.7 ± 2.7 years in the simple group. The complex group patients mostly suffered from nosocomial infection, and the simple group patients mostly had community-acquired infection (Table 1). ACS included neurosurgery (22/46), congenital developmental abnormalities (10/46), craniocerebral trauma (8/46), and acquired brain parenchymal abnormalities (8/46). The condition in 13 (6.3%) patients was accompanied by systemic diseases other than those of the nervous system. Four patients in the complex group had congenital heart disease (2/4) and received immunosuppressive therapy (2/4). Nine patients in the simple group had congenital heart disease (6/9) and congenital immunodeficiency disease (2/9) and received immunosuppressive therapy (1/9).

**Table 1:** Demographic and clinical characteristics of the study population.

Variable	Complex group (*n* = 46)	Simple group (*n* = 161)	*χ*^2^/*t*/*Z*	*p-*Value
Male (*n*)	22 (47.8%)	89 (55.3%)	0.799	0.371
Age (years)	4.0 ± 3.8	1.7 ± 2.7	−4.999	0.001*
Infection (*n*)			75.808	0.001*
Community-acquired	21 (45.6%)	156 (96.9%)		
Nosocomial	25 (54.3%)	5 (3.1%)		
Highest fever temperature (°C)	39.3 (39.0, 39.7)	39.4 (39.0, 40.0)	−0.148	0.882
Duration of fever (day)	6.5 (4.0, 14.0)	5.0 (2.0, 12.0)	−2.171	0.03*
Duration of hospitalization (day)	26.0 (15.0, 37.5)	19.0 (13.0, 31.0)	−1.999	0.046*
PCIS	4 (0, 8)	5 (3, 9)	−2.824	0.05
PRISM III	90 (84, 96)	88 (82, 90)	−1.608	0.108
Glasgow Coma Scale Score (*n*)	12 (6, 15)	14 (7, 15)	−1.467	0.142
15	15 (32.6%)	81 (50.3%)		
13–14	6 (13.0%)	9 (5.6%)		
9–12	5 (10.9%)	25 (15.5%)		
3–8	20 (43.5%)	46 (28.6%)		
Duration of coma (day)	6.0 (3.0, 9.5)	6.0 (3.0, 11.75)	0.003	0.997
Convulsion (day)	30 (65.2%)	105 (65.2%)	0	1.0
Duration of convulsion (day)	2.0 (1.0, 3.0)	2.0 (1.0, 5.5)	1.106	0.269
Cerebral dysfunction (*n*)	31 (67.4%)	80 (49.9%)	4.508	0.034*
Brain herniation (*n*)	5 (10.9%)	19 (11.8%)	0.030	0.862
Respiratory failure (*n*)	17 (36.9%)	51 (31.7%)	0.452	0.501
MODS (*n*)	0 (0%)	15 (9.3%)	4.621	0.032*
Septic shock (*n*)	1 (2.2%)	15 (9.3%)	2.559	0.110
Site of infection (*n*)			14.999	0.005*
0	29 (63.0%)	56 (34.8%)		
1	15 (32.6%)	65 (40.4%)		
2	2 (4.3%)	35 (21.7%)		
3	0 (0%)	4 (2.5%)		
4	0 (0%)	1 (0.6%)		
Outcomes (*n*)			11.038	0.004*
Normal	6 (13.0%)	57 (35.4%)		
Neurological sequelae	37 (80.4%)	86 (53.4%)		
Death	3 (6.5%)	18 (11.2%)		
Positive blood culture (*n*)	6 (13.0%)	56 (34.8%)	8.059	0.005*
Positive CSF culture (*n*)	29 (63.0%)	86 (53.4%)	1.343	0.247
CSF analysis				
Leukocyte count (×10^6^/L)	765 (158, 3,229)	627 (150, 223)	−0.406	0.685
Glucose level (mmol/L)	1.85 (0.67, 3.14)	2.09 (0.28, 3.21)	−0.409	0.683
Chlorine level (mmol/L)	118.9 (112.9, 122.8)	117.7 (113.5, 120.5)	−0.790	0.43
Protein level (g/L)	1.41 (0.84, 2.52)	1.75 (0.99, 3.65)	−1.456	0.146
Blood routine analysis				
Leukocyte count (×10^6^/L)	13.5 (10.4, 18.2)	11.5 (6.1, 17.8)	−1.934	0.053
Neutrophil percentage (%)	74.9 (56.4, 84.1)	71.4 (56.1, 85.7)	−0.706	0.48
Neutrophil count (×10^9^/L)	10.8 ± 5.7	8.0 (3.5, 12.7)	−2.065	0.039*
Platelet count (×10^9^/L)	354.4 ± 184.9	247.0 (157.0, 397.0)	−2.361	0.018*
Hemoglobin (g/L)	103.0 (91.0, 120.0)	98.0 (87.0, 113.0)	−1.635	0.102
C-reactive protein (mg/L)	78.7 (20.4, 133.0)	132.0 (65.5, 228.0)	−2.914	0.004*
Procalcitonin (ng/mL)	1.35 (0.54, 9.27)	11.65 (1.59, 50.16)	−3.548	0.001*
Interleukin 6 (pg/mL)	47.2(23.8, 352.1)	517.1(66.8, 2,276.0)	−2.868	0.004*

PCIS, pediatric clinical illness score; PRISM, pediatric risk of mortality; MODS, multiple organ dysfunction syndrome; CSF, cerebrospinal fluid.

*p < 0.05 denotes statistical significance.

### 3.2. Clinical manifestations

The clinical presentations of these patients are summarized in Table 1. Fever was the most predominant symptom in all patients, and the condition in 122 (58.9%) patients was complicated by other systemic infections. A total of 95 patients (45.9%) had pulmonary infection, 62 (30.0%) had positive blood culture, four (1.9%) had abdominal infection, three (1.4%) had urinary tract infection, two (1.0%) had skin infection, and one (0.5%) had osteomyelitis. The complex group patients had a longer duration of fever and the length of hospital stay was also longer. In this group, cerebral dysfunction (*n* = 31, 67.4%) was the leading presentation of neurological symptoms and 30 patients (65.2%) developed seizures. Brain herniation, MODS, and shock occurred more frequently in the simple group patients than in the complex ones.

### 3.3. Etiology and auxiliary examination

CSF and blood cultures were taken in 207 children, of which 125 (60.4%) were found positive, and 131 bacterial strains were detected. Five patients had mixed infection, all of which were detected in the complex group. Three of these five patients were coinfected with two types of bacteria, one with mixed four types of bacteria and one child had bacterial and *Candida albicans* infection. In the complex group, 27 patients (58.7%) tested positive in CSF culture. Moreover, 98 (60.9%) tested positive in the simple group, of which 12 had a positive blood culture. The pathogens are listed in Table 2. A total of 113 bacterial strains with positive bacterial culture and drug susceptibility analysis were analyzed. Among the 32 strains in the complex group, 20 (62.5%) were MDR bacteria, with 14 MDR gram-positive bacteria, three MDR gram-negative bacteria, and three XDR bacteria. The proportion of globate and bacilliform germ cells was 14:6. Among the 81 strains in the simple group, 45 (55.6%) were MDR bacteria, with 32 gram-positive bacteria and 13 gram-negative bacteria. The proportion of globate and bacilliform germ cells was 32:13. In the simple group, four patients on whom CSF culture was taken tested positive two or more times, and there were 12 such patients in the complex group.

**Table 2:** Microbiological findings of culture (strain).

Pathogen	Complex group (*n* = 33), *n* (%)	Simple group (*n* = 98), *n* (%)	Total (*n* = 131), *n* (%)
Gram-positive bacteria			
*Streptococcus*	9 (27.3)	65 (66.3)	74 (56.5)
*Streptococcus pneumoniae*	5 (9.6)	44 (27.3)	49 (23.0)
*Streptococcus agalactiae*	1 (1.9)	19 (11.8)	20 (9.4)
*Streptococcus pyogenes*	0 (0)	1 (0.6)	1 (0.5)
*Streptococcus constellatus*	1 (1.9)	0 (0)	1 (0.5)
*Streptococcus anginosus*	1 (1.9)	0 (0)	1 (0.5)
*Streptococcus intermedius*	1 (1.9)	0 (0)	1 (0.5)
*Streptococcus gallolyticus*	0 (0)	1 (0.6)	1 (0.5)
*Enterococcus*	7 (21.2)	3 (3.1)	10 (7.6)
*Enterococcus faecalis*	3 (5.8)	2 (1.2)	5 (2.3)
*Enterococcus faecium*	4 (7.7)	1 (0.6)	5 (2.3)
*Staphylococcus*	6 (18.2)	1 (1.0)	7 (5.3)
*Staphylococcus aureus*	1 (1.9)	1 (0.6)	2 (0.9)
*Staphylococcus epidermidis*	5 (9.6)	0 (0)	5 (2.3)
Others	1 (3.0)	5 (5.1)	6 (4.6)
*Listeria monocytogenes*	1 (1.9)	0 (0)	1 (0.5)
Unspecified	0 (0)	5 (3.1)	5 (2.3)
Gram-negative bacteria			
*Enterobacter*	3 (9.1)	18 (18.4)	21 (16.0)
*Escherichia coli*	0 (0)	15 (9.3)	15 (7.0)
*Klebsiella pneumoniae*	1 (1.9)	2 (1.2)	3 (1.4)
*Enterobacter aerogenes*	1 (1.9)	0 (0)	1 (0.5)
*Salmonella*	0 (0)	1 (0.6)	1 (0.5)
*Morganella morganii*	1 (1.9)	0 (0)	1 (0.5)
Non-fermenting	7 (21.2)	1 (1.0)	8 (6.1)
*Acinetobacter baumannii*	5 (9.6)	0 (0)	5 (2.3)
*Stenotrophomonas maltophilia*	2 (3.8)	0 (0)	2 (0.9)
*Pseudomonas aeruginosa*	0 (0)	1 (0.6)	1 (0.5)
Others	0 (0)	5 (5.1)	5 (3.8)
*Enterobacter aerogenes*	0 (0)	3 (1.9)	3 (1.4)
*Neisseria meningitidis*	0(0)	1(0.6)	1(0.5)
*Elizabethkingia meningitidis*	0(0)	1(0.6)	1(0.5)

**Figure 2:**
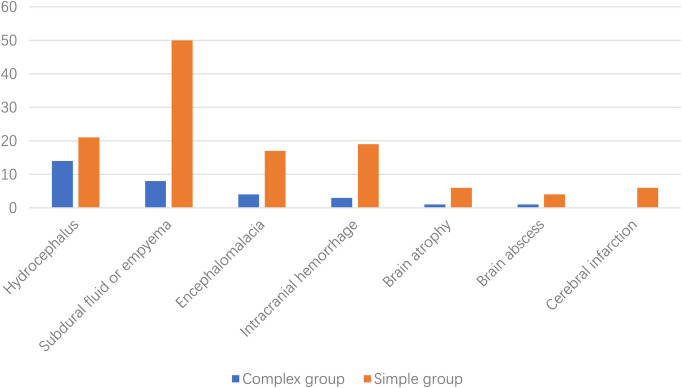
New or exacerbated abnormalities found after neuroimaging of bacterial meningitis.

Peripheral blood C-reactive protein (CRP), procalcitonin, and interleukin-6 levels in the simple group increased significantly (*p *< 0.05, Table 1). After BM, hydrocephalus and subdural or empyema were the most frequently new or exacerbated abnormalities found in neuroimaging in the complex and simple groups, respectively (Figure 2).

### 3.4. Treatment

A total of 207 (100%) patients were treated with antibiotics, and in 186 (89.8%), two or more antibiotics were combined, and the application rates of Ceftriaxone, Vancomycin, Linezolid, and Meropenem were high. The usage rates of Meropenem were higher in the complex group, whereas Ceftriaxone was more frequently used in the simple group (Table 3). The complex group used more antibiotics before the pathogen was identified, whereas the simple group performed better on de-escalating treatment after the pathogen was identified (Table 4).

**Table 3:** Treatment of the study population.

	Complex group (*n* = 46)	Simple group (*n* = 161)	*χ*^2^/*Z*	*p*-Value
Antibiotic use (*n*)				
Ceftriaxone	28 (60.9%)	126 (78.3%)	5.681	0.017*
Vancomycin	30 (65.2%)	96 (59.6%)	0.469	0.493
Linezolid	21 (45.7%)	66 (41.0%)	0.319	0.572
Meropenem	29 (63.0%)	67 (41.6%)	6.606	0.010*
Duration of antibiotic use (day)				
Ceftriaxone	8.5 (3.25, 14.5)	14.0 (6.0, 20.0)	2.283	0.022*
Vancomycin	7.5 (3.0, 15.0)	11.0 (5.0, 15.0)	0.855	0.393
Linezolid	11.0 (7.0, 23.5)	9.0 (4.75, 14.25)	−1.545	0.122
Meropenem	10.0 (6.0, 15.0)	6.0 (3.0, 11.0)	−2.380	0.017*
Mechanical ventilation (*n*)	16 (34.8%)	52 (32.3%)	0.100	0.752
Duration of ventilation (day)	5.5 (3.0, 8.0)	6.0 (2.0, 9.0)	0.109	0.913
Blood purification (*n*)	0 (0%)	5 (3.1%)	1.464	0.226
Corticosteroids (*n*)	19 (41.3%)	111 (68.9%)	11.700	0.001*
Intrathecal antibiotics (*n*)	3 (6.5%)	30 (18.6%)	3.917	0.048
CSF drainage (*n*)	23 (50.0%)	25 (15.5%)	23.879	0.001*

CSF, cerebrospinal fluid.

*p < 0.05 denotes statistical significance.

**Table 4:** Types of combined antibiotics used in children with identified pathogens (*n*).

	Complex group (*n* = 27), *n* (%)	Simple group (*n* = 98), *n* (%)	*χ* ^2^	*p* value
Before the identified pathogen (*n*)			9.641	0.022*
1	3 (11.1)	6 (6.1)		
2	14 (51.9)	79 (80.6)		
3	9 (33.3)	12 (12.2)		
4	1 (3.7%	1 (1.0)		
After the identified pathogen (*n*)			6.630	0.036*
1	5 (18.5)	23 (23.5)		
2	17 (62.9)	71 (72.4)		
3	5 (18.5)	4 (4.1)		

*p < 0.05 denotes statistical significance.

Moreover, 68 out of 207 (32.8%) patients required mechanical ventilation, and 130 (62.8%) used corticosteroids. A total of 33 (15.9%) patients were treated with intrathecal antibiotics, and 48 (23.2%) underwent CSF drainage. The number of complex group patients who were given steroid (41.3% vs. 68.9%) and intrathecal injections was lower (6.5% vs. 18.6%; Table 3). The number of complex group patients who received CSF drainage was higher (50% vs. 15.5%; Table 3).

### 3.5. Outcomes

**Figure 3:**
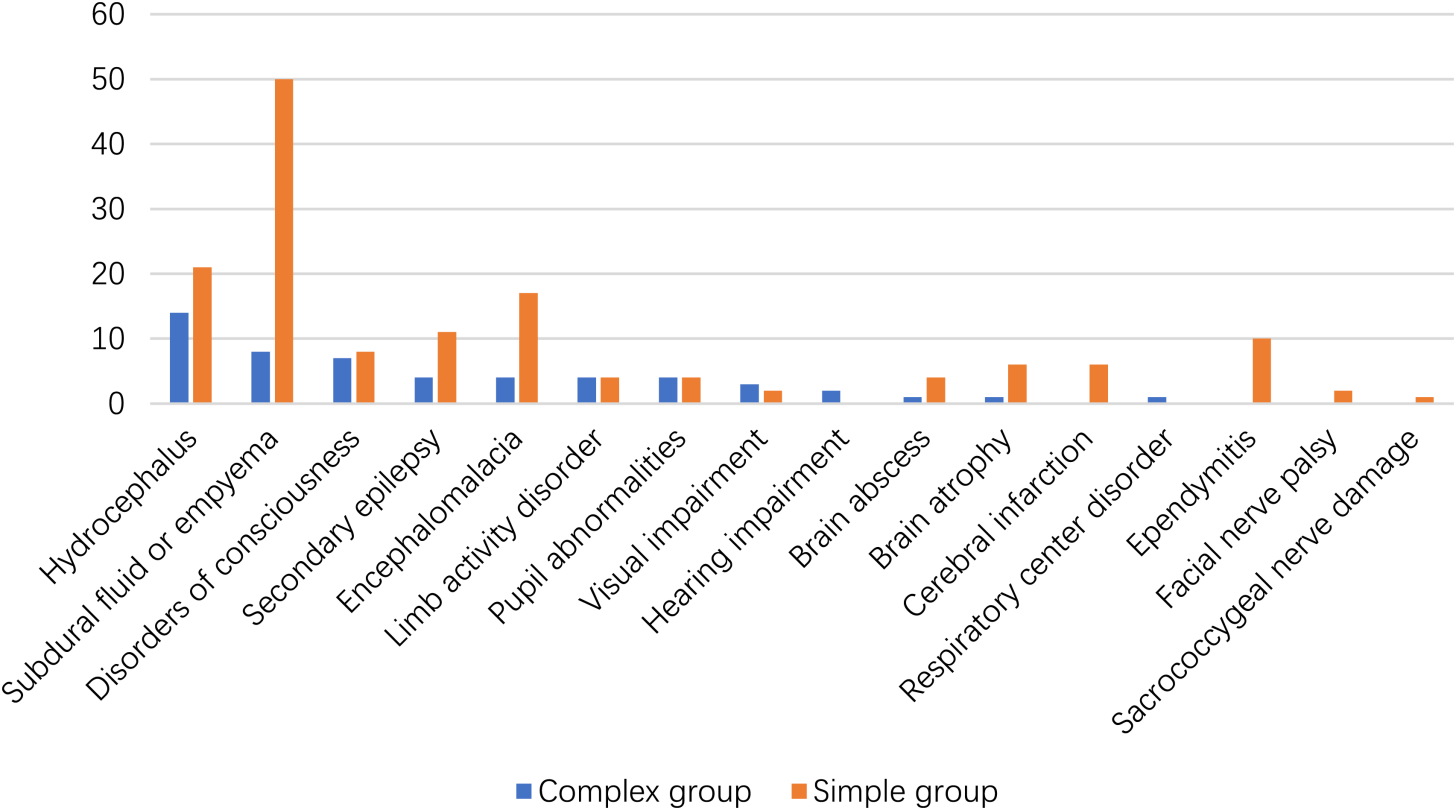
The neurological sequelae of the surviving children.

The primary outcomes were classified as death, presence of neurological sequelae, and absence of sequelae. The classification of neurological sequelae is shown in Figure 3. Children in the complex group had higher neurological sequelae (80.4% vs. 53.4%, Table 1). However, the complex group had a lower in-hospital mortality rate. There was no difference in the in-hospital mortality rates of the two groups. Moreover, 11 and 27 patients in the complex and simple groups had two or more neurological sequelae, respectively.

Among the 207 patients, 21 (10.1%) died, and of these, 19 (90.5%) had a positive CSF culture and 10 (47.6%) had a positive blood culture. There were three deaths in the complex group, including two nosocomial infections, and all of them had septic shock. There were 18 deaths in the simple group, of which 17 had community-acquired infection, and 8 also had septic shock and another 8 also MODS.

## 4. Discussion

Community-acquired BM is associated with an abnormal blood–brain barrier and a strong invasive ability of bacteria, and most patients experience immune function changes (2). Nosocomial meningitis is mostly caused by postneurosurgery and head trauma (10). Our study shows that in community-acquired BM, there are 11.8% of patients with ACS; the previous study reported that 3% had anatomic defects of CSF leakage in community-acquired BM (19). It has been found that ACS is a risk factor for community-acquired BM (2, 6). ACS is also a risk factor for recurrent BM (20). Clinicians are highly aware of the occurrence of meningitis in children with ACS. Most of these children are diagnosed using CSF tests when they develop fever, but they may not have neurological manifestations. The PCIS and PRISM showed that the condition of simple group patients was more severe. We found that infections in children with ACS were limited, and 60% of them had only neurological infections. Bloodstream infections are rare, and no cases of MODS have been reported. The positivity rate of CSF culture in patients with ACS is higher, which may be attributed to bacteria entering the brain more easily, a large bacterial invasion, and an evident colonization state. Positive blood culture did result in group bias. But we reasoned that this bias cannot be completely avoided in clinical work. This is consistent with the local bacterial invasion after the disruption of the external barrier (14). In contrast, children with NCS mostly experienced bloodstream infection, with nearly 10% of children having MODS. This may be attributed to the fact that sustained bacteremia is important for the development of BM when bacteria reach the leptomeninges via the bloodstream (2). High bacteria loads give them the ability to escape from host defenses and reach the threshold level of bacteremia that is necessary for invading the meninges (15). This is borne out by the fact that the simple group patients in this study had a more positive blood culture. Septic shock caused by bloodstream infection leads to a high early mortality rate, and the high overall mortality rate in the simple group may also be related to more frequent bloodstream infections. Savonius et al. found that CSF bacterial counts were related to the outcomes of BM (21). We found that the concentrations of CRP, procalcitonin, and interleukin-6 increased in both groups, and the increase was more significant in the simple group than in the complex group, which was associated with severe bloodstream infection.

In clinical treatment, the key to improving prognosis is to select antibiotics that penetrate the meninges and are effective against possible bacteria, as early as possible. One study showed that delaying antibiotic use for more than 6 h would lead to an 8.4-fold increased risk of death (22). ACS is mostly acquired; it can result from congenital abnormalities of the skull, or it can result after neurosurgery, ear–nose–throat surgery, or neurotrauma, or trauma (23, 24). BM with ACS mostly occurs during hospitalization (10, 14, 25, 26). We identified multiple cases of drug-resistant coagulase-negative *Staphylococcus*, *Streptococcus intermedius*, and XDR *A. baumannii* in the complex group. The mortality rate increases when XDR infection occurs in the CNS (27). Compared with that of the NCS, the main difference among the pathogens of children with ACS was that nosocomial infection and MDR bacteria were more common. The proportion of antibiotic types and combinations was high because of the empirical coverage of these pathogens. Therefore, the usage rate and course of Meropenem were also significantly greater in those with ACE than in those with NCS, which is in line with the recommendations of most guidelines (14). Although most cases of children having BM with NCS were community-acquired, most of them showed severe infection and shock when they were admitted to the hospital. There were also many types of antibiotics to cover possible pathogens in the early stage. However, most of them did not cover drug-resistant strains but covered possible pathogenic bacteria; thus, the proportion of targeted treatment in these children was higher after the pathogen was identified.

The drainage rate of children with ACS was significantly higher than that of those with NCS. Drainage was done for treating other craniocerebral diseases. However, ACS combined with BM can lead to intracranial empyema, and drainage is usually performed for intraventricular empyema. Drainage in children with NCS was mainly done to treat severe subdural empyema (28). Children with NCS used more hormones, which may be related to severe systemic inflammatory response, MODS, and shock. We found that intrathecal injections were used according to the guidelines (29) for the treatment of XDR *A. baumannii* meningitis in children with ACS. However, more intrathecal antibiotics were given for the simple group patients who did not have *A. baumannii* infections. They were given to produce higher concentrations in the CSF rather than yielding them through intravenous administration. As shown in the previous article, the intrathecal injection of antibiotics is an alternate route for treating CNS infections, and direct access to the CSF space via intrathecal or intraventricular daptomycin instillation provides an alternate route of administration that has proved highly effective (30).

We found that preschool children comprised many patients with BM, but children having BM with ACS were older than those with NCS, which may be related to the age of onset of ACS. This is in contrast to the results of a previous study on BM, wherein a high incidence rate of BM with ACS was observed in children aged younger than 2 years (31). The overall incidence rate of neurological sequelae was nearly 60%, and the mortality rate was 10%, which was higher than that reported in a previous study on community-acquired BM (32). The high incidence of sequelae may be related to the research background of the PICU, which had a history of patients with severe brain injury, bloodstream infection, and more cases of MODS and shock. The early neurological sequelae of BM included hydrocephalus, ependymitis, and subdural effusion, whereas the long-term sequelae included epilepsy and hearing loss (23). In this study, the sequelae of children with ACS were more likely to be related to primary craniocerebral dysplasia or secondary craniocerebral trauma and neurosurgery, and the effect of intracranial infection on sequelae needs to be further studied. Children with ACS had a higher proportion of new-onset or worsening hydrocephalus after the acute phase on head neuroimaging than in those of the simple group, a finding similar to those of other related studies (33, 34). In a targeted analysis of 21 patients who died, approximately half of these deaths were related to cerebral functional failure, which was similar to Sharew et al.'s finding that complications of cerebral functional failure accounted for 43% of deaths (35). Intracranial pressure mostly increased in the early course of the disease, and in children, it reached its peak at 36 h (36). The GCS score of patients with ACS was mostly 3–8 points, and some children with ACS had undergone brain surgery and experienced brain trauma and brain parenchyma injury. Moreover, the disturbance of consciousness was related not only to infection but also to the primary disease and its treatment. Although there were differences in severe disturbance of consciousness between the two groups, there were no significant differences in other clinical manifestations of the nervous system. This may be related to drainage. Drainage reduces intracranial hypertension by transferring the CSF (37). Even in instances of severe cell damage and intracranial pressure changes, drainage could serve as a protection to the brain.

## 5. Conclusion

Children who had BM with ACS tended to belong to the older age group and had fewer bloodstream infections, lower mortality rates, and higher incidence rates of neurological sequelae. Pathogens of BM with ACS are more likely nosocomial and MDR bacteria. Early treatment and prevention of BM with ACS are significantly important.

## Data Availability

The raw data supporting the conclusions of this article will be made available by the authors without undue reservation.
